# SIRT1 rs10823108 and FOXO1 rs17446614 responsible for genetic susceptibility to diabetic nephropathy

**DOI:** 10.1038/s41598-017-10612-7

**Published:** 2017-08-31

**Authors:** Yanyan Zhao, Junfang Wei, Xuefeng Hou, Huimiao Liu, Feng Guo, Yingni Zhou, Yuanyuan Zhang, Yunhui Qu, Junfei Gu, Yuanli Zhou, Xiaobin Jia, Guijun Qin, Liang Feng

**Affiliations:** 1 Division of Endocrinology, Department of Internal Medicine, The First Affiliated Hospital, Zhengzhou University, Zhengzhou, 450052 China; 2 Key Laboratory of New Drug Delivery System of Chinese Materia Medica, Jiangsu Provincial Academy of Chinese Medicine, Nanjing, 210028 China; 3 The fifth affiliated hospital of Zhengzhou University, Zhengzhou, 450052 China; 4 Department of Clinical Laboratory, The First Affiliated Hospital of Zhengzhou University, Zhengzhou, 450052 China

## Abstract

SIRT1 and FOXO1 play an important role in the pathogenesis of diabetic nephropathy (DN). However, the association between genetic polymorphisms and susceptibility to type 2 DN (T2DN) has not been explored. In this study, a total of 1066 patients with type 2 diabetes mellitus (T2DM) (413 without and 653 with DN) were enrolled. The genotypes of three htSNPs (rs3818292, rs4746720, rs10823108) within SIRT1 and two htSNPs (rs2721068, rs17446614) in FOXO1 were determined by PCR-RFLP. HbA1C, LDL, HDL, TC, and TG levels were also examined. SIRT1 rs10823108 AA genotype was significantly associated with a decreased risk of DN (OR = 0.60, 95%CI: 0.38–0.97), while GA genotype (OR = 1.77, 95%CI: 1.33–2.35) and AA genotype (OR = 2.32, 95%CI: 1.25–4.34) of FOXO1 rs17446614 was associated with an increased T2DN risk. The interactions among rs1744 6614, BMI and duration of diabetes (OR: 2.63, 95%CI: 1.23–4.31) were also observed. Subsequent haplotype analysis revealed that two haplotype defined by AC (OR: 1.50, 95%CI: 1.15–1.94) and AT (OR: 1.79, 95%CI: 1.06–2.80) within FOXO1 gene may increase the risk of T2DN. In conclusion, genetic variant rs10823108 in SIRT1 and variant rs17446614 in FoxO1 may contribute to the risk of DN in T2DM patients.

## Introduction

Diabetic nephropathy (DN) is one of the late complication of diabetes that affects approximately 40% of all patients with diabetes, irrespective of glycemic control^1, 2^ and remains the leading cause of end-stage renal disease (ESRD) in the United States^3^, and in China^4^. Mounting evidence has showed that the pathogenesis of DN was associated partially with a prolonged duration or inadequate metabolic and/or blood pressure control in some cases. However, a clinically significant phenomenon can be observed that even diabetic individuals with excellent blood glucose control may still develop renal complications. The vast majority of the variability in incident nephropathy remains unaccounted for by conventional risk factors. Whereas, investigations on the familial clustering of DN in T2DM and the heritability of DN and its related traits provide compelling evidence that genetic factors contribute to its susceptibility^5–7^.

The role of Forkhead box O1 (FOXO1), caused more attention in DN, due to its important role in oxidative stress resistance^8, 9^. It has been suggested that FOXO1 is closely related to the pathogenesis of DN^10^. There is emerging evidence that suggests repressing FoxO1 contribute to adipogenesis in porcine pre-adipocyte^11^. It is also interesting that SIRT1 regulates adipocyte differentiation through FoxO1 acetylation/deacetylation, although the mechanism is not understood^12^. Silent mating type information regulation 2 homolog 1 (SIRT1), an NAD^+^ dependent deacetylase, may regulate multiple cellular functions, including apoptosis, mitochondrial biogenesis, inflammation, glucose/lipid metabolism, autophagy, blood pressure and sodium balance, through the deacetylation of target proteins^13, 14^. The activation of SIRT1 in the kidney may be a new therapeutic target to increase resistance to many causal factors in the development of renal diseases, including DN^15^. Our previous study suggested that the FoxO1 activity was significantly reduced and with a concomitant decrease in the expression of FoxO1 target gene, catalase in diabetic kidney^16^. The FoxO1 downregulation correlated with an increase in the generation of malondialdehyde (MDA), a decrease in the activity of SOD and an increase in the expression of collagen IV and fibronectin proteins in renal cortex^10^. These results indicate that SIRT1/FOXO1 axis-mediated signaling pathway may be considered as a potential therapeutic target for DN. Therefore, SIRT1/FOXO1 are a very plausible candidate gene potentially contributing to the genetic polymorphism of DN. The limited finding identified 4 SNPs and haplotype consisting of the 11 SNPs within SIRT1 locus were nominally or significantly associated with DN^17^. Meanwhile,FOXO1 gene has been found to be involved in the susceptibility to type 2 diabetes in German^18^. However, it has not been reported that whether SIRT1 or FOXO1 genetic polymorphism was associated with DN in Chinese population.

In order to explore the genetic role of SIRT1 and FOXO1, and environmental interactions in diabetic nephropathy susceptibility in Chinese Han subjects with T2DN, we investigated the association between single nucleotide polymorphisms (SNPs) within the SIRT1 and FOXO1 gene and DN in Chinese Han subjects.

## Results

### Demographic and clinical characteristics of study samples

Baseline characteristics (including sociodemographic factors, diabetes related variables, lifestyle behaviors, biochemical variables, and history of diseases) of study subjects (653 T2DN cases and 413 T2DM without DN controls) were illustrated in Table 1. The mean age among cases (62.46 ± 11.04) was significantly older than that of controls (58.35 ± 12.07). The mean durations of diabetes were significantly different between cases (11.54 ± 7.47) and controls (14.29 ± 4.95). T2DN cases had significant higher BMI, HbA1C, total cholesterol level, and lower HDL-C level than controls. In addition, increased risks of T2DN were associated with smoking (OR = 1.40, 95% CI:1.04, 1.88) and hypertension (OR = 1.95, 95% CI:1.51, 2.50).

**Table 1:** Demographic and clinical characteristics of study samples.

Characteristics		Case (n = 653)	Control (n = 413)	P	OR
**Sociodemographic Factors**					
Gender	male	413(63.25%)	255(61.74%)		1
	female	240(36.75%)	158(38.26%)	0.62	0.94(0.73, 1.21)
Age (years)		62.46 ± 11.04	58.35 ± 12.07	< 0.01	
**Diabetes-related Variables**					
Duration of diabetes (years)		11.54 ± 7.47	14.29 ± 4.95	< 0.01	
BMI (kg/m^2^)		26.19 ± 2.55	25.25 ± 2.74	< 0.01	
HbA1C (%)		8.88 ± 2.23	8.54 ± 1.74	< 0.01	
**Lifestyle behaviors**					
Smoking	no	483(73.97%)	330(79.90%)		1
	yes	170(26.03%)	83(20.10%)	0.03	1.40(1.04, 1.88)
Drinking	no	553(84.69%)	361(87.41%)		
	yes	100(15.31%)	52(12.59%)	0.22	1.26(0.88, 1.80)
**Biochemical variables**					
Total cholesterol (mmol/L)		4.40 ± 1.39	4.23 ± 1.09	0.03	
Triglycerides (mmol/L)		1.87 ± 1.40	1.80 ± 1.80	0.47	
HDL-C (mmol/L)		1.06 ± 0.33	1.13 ± 0.35	< 0.01	
LDL-C (mmol/L)		2.65 ± 1.00	2.55 ± 0.87	0.11	
**History of diseases**					
Hypertension	no	231(35.38%)	213(51.57%)		1
	yes	422(64.62%)	200(48.43%)	< 0.01	1.95(1.51, 2.50)
DM Family history	no	414(63.40%)	259(62.71%)		1
	yes	239(36.60%)	154(37.29%)	0.82	0.97(0.75, 1.25)

### Effects of htSNPs in SIRT1, FOXO1 and T2DN risk

No significant deviations from Hardy-Weinberg equilibrium for the five polymorphisms was found among the 413 controls (P > 0.05). The results of single locus analysis revealed that AA genotype carriers of SIRT1 gene locus rs10823108 had decreased risk of suffering from DN (OR = 0.60, 95%CI: 0.38–0.97) than GG genotype carriers. And the decreased risk were still significant in the recessive genetic model (OR = 0.58, 95%CI: 0.37–0.91). GA genotype (OR = 1.77, 95%CI: 1.33–2.35) and AA genotype (OR = 2.32, 95%CI: 1.25–4.34) of FOXO1 gene locusrs17446614 had increased risk of suffering from T2DN than GG genotype carriers. The significant association between T2DN risk and rs17446614 were significant in both dominant genetic model (OR = 1.86, 95%CI: 1.42–2.43) and recessive genetic model (OR = 1.96, 95%CI: 1.06–3.63). After being adjusted for age, gender, duration of diabetes, BMI, HbA1C, total cholesterol, triglycerides, HDL-C, LDL-C, hypertension, DM family history, smoking and drinking status, all the associations above were still significant. However, no significant difference was observed in the frequency distribution of rs4746720, rs3818292 and rs2721068 polymorphisms between T2DN patients and controls (Table 2).

**Table 2:** Genotype among cases and controls and their association withT2DN risk.

SNP	Case(%)	Control(%)	P^a^	OR(95%CI)^b^	P^b^	OR(95%CI)^c^	P^c^
rs3818292							
AA	360(55.13)	226(54.72)		1		1	
AG	242(37.06)	146(35.35)		1.04(0.80, 1.36)	0.770	1.01(0.76, 1.35)	0.940
GG	51(7.81)	41(9.93)	0.26	0.78(0.50, 1.22)	0.270	0.69(0.43, 1.11)	0.120
AG + GG	293(44.87)	187(45.28)		0.98(0.77, 1.26)	0.900	0.94(0.72, 1.23)	0.650
AA + AG	602(92.29)	372(90.07)		1		1	
GG	51(7.81)	41(9.93)		0.77(0.50, 1.18)		0.70(0.44, 1.11)	0.120
A	962(73.66)	598(72.40)		1			
G	344(26.34)	228(27.60)		0.94(0.77, 1.14)	0.520		
rs4746720							
TT	191(29.25)	121(29.30)		1		1	
CT	331(50.69)	210(50.85)		0.99(0.75, 1.33)	0.990	0.90(0.66, 1.23)	0.500
CC	131(20.06)	82(19.85)	0.60	1.01(0.71, 1.45)	0.950	1.01(0.69, 1.46)	0.990
CT + CC	462(70.75)	292(70.70)		0.98(0.77, 1.26)	0.910	0.92(0.69, 1.23)	0.990
TT + CT	522(79.94)	331(80.15)		1		1	
CC	131(20.06)	82(19.85)		1.01(0.75, 1.38)	0.940	1.01(0.72, 1.40)	0.970
T	713(54.59)	452(54.72)		1			
C	593(45.41)	374(45.28)		1.00(0.84, 1.20)	0.950		
rs10823108							
GG	353(54.06)	224(54.23)		1		1	
GA	261(39.97)	148(35.84)		1.12(0.86, 1.45)	0.400	1.07(0.81, 1.43)	0.620
AA	39(5.97)	41(9.93)	0.36	0.60(0.38, 0.97)	0.040	0.57(0.34, 0.94)	0.030
GA + AA	300(45.94)	189(45.77)		1.01(0.79, 1.29)	0.950	0.97(0.74, 1.27)	0.820
GG + AG	614(94.03)	372(90.07)		1		1	
AA	39(5.97)	41(9.93)		0.58(0.37, 0.91)	0.020	0.56(0.34, 0.92)	0.020
G	967(74.04)	596(72.15)		1			
A	339(25.96)	230(27.85)		0.91(0.75, 1.11)	0.340		
rs17446614							
GG	392(60.03)	304(73.61)		1		1	
GA	219(33.54)	95(23.00)		1.77(1.33, 2.35)	< 0.001	1.54(1.14, 2.13)	< 0.001
AA	42(6.43)	14(3.39)	0.07	2.32(1.25, 4.34)	0.02	2.34(1.77, 4.13)	< 0.001
GA + AA	261(39.97)	109(26.39)		1.86(1.42, 2.43)	< 0.001	1.65(1.19, 2.26)	< 0.001
GG + AG	611(93.57)	399(96.61)		1		1	
AA	42(6.43)	14(3.39)		1.96(1.06, 3.63)	0.03	2.07(1.38, 4.14)	< 0.001
G	1003(76.80)	703(85.11)		1			
A	303(23.20)	123(14.89)		1.73(1.37, 2.18)	< 0.001		
rs2721068							
TT	77(11.79)	40(9.68)		1		1	
CT	296(45.33)	192(46.49)		0.80(0.53, 1.22)	0.300	0.64(0.40, 1.02)	0.060
CC	280(42.88)	181(43.83)	0.29	0.81(0.53, 1.23)	0.310	0.73(0.46, 1.15)	0.180
CT + CC	576(88.21)	373(90.32)		0.80(0.54, 1.20)	0.280	0.68(0.44, 1.05)	0.080
TT + CT	373(57.12)	232(56.17)		1		1	
TT	77(11.79)	40(9.68)		0.97(0.75, 1.23)	0.760	0.95(0.73, 1.24)	0.690
T	450(34.46)	272(32.93)		1			
C	856(65.54)	554(67.07)		0.93(0.78, 1.12)	0.470		

^a^P value of Hardy–Weinberg equilibrium in Controls.

^b^Chi square test for genotype distributions between cases and controls.

^c^Data were calculated by logistic regression analysis with adjusted for age, gender,duration of diabetes, BMI, HbA1C, total cholesterol, triglycerides, HDL-C, LDL-C,history of Hypertension, DM family history, smoking and drinking.

### Stratified analysis of SNP genotypes and T2DN risk

We further evaluated effects of the five htSNPs stratified by age, gender, duration of diabetes, BMI, HbA1C, total cholesterol, triglycerides, HDL-C, LDL-C, history of hypertension, DM family history, smoking and drinking status. As indicated in Table 3, a significant increased risk of T2DN was observed in subjects with TC level > 5 (OR: 2.11; 95%CI: 1.06–4.21) and carried variant genotypes of rs3818292. TC + CC genotypes of rs2721068 had a significantly decreased risk of T2DN among male subjects (OR: 0.47; 95%CI: 0.25–0.87), younger subjects (OR: 0.35; 95%CI: 0.17–0.71), subjects with BMI ≥ 24(OR: 0.46; 95%CI: 0.26–0.79), LDL-C ≤ 3.5(OR: 0.61; 95%CI: 0.38–0.98) and smoking (OR: 0.14; 95%CI: 0.03–0.64). The increased T2DN risk of rs17446614 GA + AA genotype was still significant in gender, age, BMI, TC, hypertension, DM family history and smoking subgroups. And GA + AA genotypes of rs17446614 had a significantly increased risk of T2DN among subjects with HbA1C > 8 (OR: 1.48; 95%CI: 0.99–2.23), subjects with LDL-C ≤ 3.5 (OR: 2.21; 95%CI: 1.60–3.06).

**Table 3:** Stratification analysis of the five SNPs polymorphisms and DN susceptibility.

Variables	rs3818292 AG + GG /AA		rs4746720 TC + CC /TT		rs10823108 GA + AA/GG		rs17446614 GA + AA/GG		rs2721068 TC + CC /TT	
	OR (95%CI)^a^	P^b^	OR (95%CI)^a^	P^b^	OR (95%CI)^a^	P^b^	OR (95%CI)^a^	P^b^	OR (95%CI)^a^	P^b^
**Gender**										
male	0.76(0.54, 1.06)	0.11	1.07(0.73, 1.55)	0.73	0.88(0.62, 1.21)	0.44	2.32(1.58, 3.41)	< 0.0001	0.47(0.25, 0.87)	0.02
female	1.32(0.84, 2.08)	0.23	0.77(0.47, 1.25)	0.29	1.06(0.67, 1.68)	0.79	2.67(1.62, 4.40)	< 0.0001	0.96(0.51, 1.82)	0.90
**Age**										
≤60	0.79(0.53, 1.16)	0.23	1.06(0.69, 1.64)	0.78	0.91(0.62, 1.34)	0.63	2.65(1.68, 4.18)	< 0.0001	0.35(0.17, 0.71)	0.00
>60	1.09(0.75, 1.57)	0.66	0.86(0.58, 1.30)	0.48	1.03(0.71, 1.49)	0.89	2.02(1.34, 3.02)	0.0007	1.03(0.57, 1.86)	0.92
**Duration of diabetes**										
≤10	1.01(0.61, 1.68)	0.97	0.95(0.57, 1.59)	0.85	1.00(0.67, 1.57)	0.99	1.03(0.63, 1.67)	0.53	0.63(0.29, 1.37)	0.25
>10	0.89(0.64, 1.26)	0.52	0.95(0.66, 1.37)	0.79	0.94(0.67, 1.32)	0.73	1.27(0.55, 2.32)	0.49	0.72(0.42, 1.24)	0.23
**BMI**										
<24	1.23(0.67, 2.28)	0.50	0.79(0.43, 1.43)	0.43	0.66(0.37, 1.16)	0.15	4.60(2.44, 8.66)	<0.0001	1.93(0.78, 4.78)	0.15
≥ 24	0.95(0.70, 1.29)	0.75	0.97(0.70, 1.36)	0.89	1.06(0.78, 1.44)	0.70	1.89(1.35, 2.65)	0.0002	0.46(0.26, 0.79)	0.01
**HbA1C**										
≤8	1.21(0.83, 1.76)	0.33	0.93(0.62, 1.41)	0.73	1.13(0.78, 1.65)	0.52	1.06(0.58, 1.39)	0.68	0.62(0.34, 1.15)	0.13
>8	0.75(0.51, 1.10)	0.14	0.88(0.58, 1.33)	0.54	0.87(0.60, 1.27)	0.47	1.48(0.99, 2.23)	0.0600	0.77(0, 42, 1.41)	0.40
**HDL-C**										
≤1	1.17(0.78, 1.74)	0.44	0.66(0.41, 1.05)	0.08	1.13(0.76, 1.68)	0.55	1.07(0.34, 1.78)	0.53	0.51(0.25, 1.05)	0.07
>1	0.83(0.57, 1.20)	0.31	1.21(0.82, 1.79)	0.36	0.88(0.61, 1.27)	0.48	1.09(0.41, 2.11)	0.78	0.81(0.46, 1.46)	0.49
**LDL-C**										
≤3.5	0.93(0.69, 1.25)	0.62	0.93(0.67, 1.28)	0.64	0.95(0.71, 1.27)	0.71	2.21(1.60, 3.06)	<0.0001	0.61(0.38, 0.98)	0.04
>3.5	0.93(0.40, 2.17)	0.86	0.87(0.35, 2.13)	0.75	0.68(0.29, 1.59)	0.38	1.45(0.96, 2.21)	0.12	1.80(0.54, 6.01)	0.34
**TC**										
≤5	0.84(0.62, 1.15)	0.28	1.03(0.72, 1.45)	0.88	0.81(0.60, 1.11)	0.20	2.29(1.62, 3.22)	<0.0001	0.70(0.41, 1.17)	0.17
>5	2.11(1.06, 4.21)	0.03	0.78(0.38, 1.60)	0.49	1.92(0.98, 3.79)	0.06	2.57(1.16, 5.70)	0.0200	0.65(0.25, 1.78)	0.40
**TG**										
≤1.65	0.99(0.69, 1.41)	0.94	1.00(0.68, 1.48)	0.98	0.94(0.66, 1.35)	0.75	1.09(0.39, 2.13)	0.76	0.67(0.38, 1.18)	0.17
>1.65	1.00(0.65, 1.54)	0.99	0.76(0.47, 1.22)	0.25	1.02(0.67, 1.57)	0.93	1.18(0.73, 2.55)	0.89	0.82(0.39, 1.69)	0.59
**Hypertension**										
No	0.94(0.63, 1.41)	0.76	0.97(0.63, 1.51)	0.90	1.01(0.67, 1.52)	0.95	2.68(1.74, 4.14)	<0.0001	0.89(0.49, 1.63)	0.71
Yes	1.00(0.70, 1.42)	0.98	0.88(0.60, 1.31)	0.53	0.95(0.67, 1.35)	0.78	2.03(1.35, 3.05)	0.0007	0.53(0.28, 1.02)	0.06
**DM Family history**										
No	0.97(0.69, 1.36)	0.86	0.93(0.64, 1.35)	0.69	1.10(0.78, 1.55)	0.58	2.27(1.55, 3.34)	<0.0001	0.50(0.28, 0.91)	0.02
Yes	0.91(0.60, 1.40)	0.68	0.92(0.57, 1.47)	0.72	0.83(0.54, 1.27)	0.38	2.33(1.45, 3.76)	0.0005	1.12(0.59, 2.14)	0.74
**Drinking**										
No	0.92(0.69, 1.23)	0.58	0.85(0.63, 1.18)	0.35	0.96(0.72, 1.28)	0.76	1.52(0.82, 2.49)	0.56	0.85(0.58, 1.33)	0.47
Yes	1.08(0.54, 2.17)	0.82	2.02(0.87, 4.67)	0.10	1.12(0.56, 2.25)	0.75	1.14(0.54, 2.40)	0.7300	0.75(0.85, 3.59)	0.13
**Smoking**										
No	0.96(0.70, 1.31)	0.79	0.84(0.60, 1.17)	0.30	0.95(0.70, 1.30)	0.74	2.35(1.66, 3.31)	<0.0001	0.88(0.55, 1.43)	0.61
Yes	0.93(0.63, 1.61)	0.79	1.12(0.61, 2.06)	0.71	1.05(0.60, 1.83)	0.87	2.06(1.10, 3.85)	0.0200	0.14(0.03, 0.64)	0.01

^a^Adjusted for age, gender,duration of diabetes, BMI, HbA1C, total cholesterol, triglycerides, HDL-C, LDL-C,history of Hypertension, DM family history, smoking and drinking (the stratified factor in each stratum excluded).

^b^ *P* value for heterogeneity test.

### Analysis of haplotype

The combined effects of the five polymorphisms on the risk of BC were evaluated by Haplotype analysis (Table 4). A_rs3818292_C_rs4746720_G_rs10823108_ of SIRT1 and G_rs17446614_C_rs2721068_ of FOXO1were the most common haplotype in cases and controls. Haplotype analysis show that two haplotypes G_rs17446614_C_rs2721068_ (OR:0.78, 95%CI: 0.65–0.93) and G_rs17446614_T_rs2721068_ (OR:0.77,95%CI: 0.63–0.93) within FOXO1 may reduce the risk of T2DN. And Carrier of A_rs17446614_C_rs2721068_ (OR: 1.50,95%CI: 1.15–1.94) and A_rs17446614_T_rs2721068_ (OR: 1.79,95%CI: 1.06–2.80) may increase the risk of T2DN. However, no association was observed for other haplotypes in SIRT1.

**Table 4:** Haplotype Analysis of five Polymorphism Sites.

Gene	Haplotype	Cases (%)	Controls (%)	χ^2^	P	OR (95%CI)
SIRT1^a^	A C A	11.89(9.0)	10.70(1.3)	0.72	0.40	0.70(0.31, 1.61)
	A C G	562.12(43.0)	343.59(41.6)	0.43	0.51	1.06(0.89, 1.27)
	A T A	29.55(2.3)	26.76(3.2)	1.88	0.17	0.69(0.41, 1.18)
	A T G	358.44(27.4)	216.95(26.3)	0.36	0.55	1.06(0.87, 1.29)
	G C A	4.44(0.3)	5.31(0.6)	1.02	0.31	0.53(0.15, 1.87)
	G C G	14.55(1.1)	14.41(1.7)	1.50	0.22	0.64(0.31, 1.32)
	G T A	293.12(22.4)	187.23(22.7)	0.01	0.90	0.99(0.80, 1.22)
	G T G	31.89(2.4)	21.05(2.5)	0.02	0.88	0.96(0.55, 1.67)
FOXO1^b^	A C	209.39(16.0)	93.44(11.3)	9.25	0.00	1.50(1.15, 1.94)
	A T	108.61(8.3)	11.56(1.4)	45.52	0.00	1.79(1.06, 2.80)
	G C	646.61(49.5)	460.56(55.8)	7.91	0.00	0.78(0.65,0.93)
	G T	341.39 (26.1)	260.44(31.5)	7.26	0.01	0.77(0.63,0.93)

^a^SNPs sequence: rs3818292,rs4746720 and rs10823108.

^b^SNPs sequence: rs17446614 and rs2721068.

The joint effect and potential locus-locus interaction T2DN risk were calculated by categorizing the SNPs (rs3818292, rs4746720, rs10823108, rs2721068, and rs17446614) into the number of combined variant alleles (Table 5). No statistical increased or decreased risk for T2DN in each subgroup was observed when compared to individuals with 0–2 mutation allele. And there was no increased dose-dependent manner on the combined effect of the five htSNPs.

**Table 5:** Combined Effect of the five SNPs on DN.

Combined SNPs	Cases (%)	Controls (%)	χ^2^	P	OR (95%CI)
0–2	123(18.84)	76(18.41)			1
3–4	340(50.07)	232(56.17)	0.35	0.56	0.91(0.65, 1.26)
5–6	170(26.03)	96(23.24)	0.22	0.64	1.09(0.75, 1.60)
≥7	20(3.06)	9(2.18)	0.55	0.46	1.37(0.60, 3.17)
			0.81^a^	0.37^a^	
Total	653	413			

^a^Trend Chi-square and P values.

### The interaction of SIRT1 and FOXO1 gene on the pathogenesis of T2DN

Multifactor Dimensionality Reduction (MDR) analysis was performed to analyze the gene-environment factors interaction with SNPs of SIRT1 and FOXO1(rs3818292, rs4746720, rs10823108, rs2721068, and rs17446614), age, gender, duration of diabetes, BMI, HbA1C, total cholesterol, triglycerides, HDL-C, LDL-C, Hypertension, DM family history, smoking and drinking status (Table 6). All demographic and clinical factors were initially dichotomized. The results of gene-environment interaction analysis presented a best model including of three factors (rs1746614, BMI, Duration of diabetes), which could increase 2.63 -fold in the T2DN risk in the ‘high risk group’ compared with the ‘low risk group’ (OR: 2.63, 95%CI: 1.23–4.31). Logistic regression analysis was used to verify the best MDR interaction model, and the results are consistent with that of MDR.

**Table 6:** Interaction results between the five SNPs and risk factors by MDR.

Model	Testing balacc	CV consistency	χ^2^	OR(95%CI)	*P*
Duration of diabetes	0.6057	10/10	4.66	1.94(1.09, 3.13)	0.03
BMI, Duration of diabetes	0.5591	5/10	6.74	2.24 (1.56, 3.63)	0.01
rs1746614, BMI, Duration of diabetes	0.6091	6/10	5.01	2.63(1.23, 4.31)	0.02

## Discussion

It has been reported that genetic predisposition is one of the main risk factors for the development of DN^19^. Evidence of familial aggregation of diabetic nephropathy in T2DM and the heritability confirm that genetic factors play a significant role in the development of DN^20, 21^. There is now an increasing body of data to suggest that genetic factors may contribute to the initiation and development of diabetic nephropathy. Nevertheless, there were only a very limited number of studies that have characterized SIRT1 genes for DN in Japanese Caucasians, while no study focused on the association between FOXO1 gene and diabetic nephropathy.

In the present study, we investigated the role of SIRT1 and FOXO1 variants and their haplotypes in T2DN among unrelated Chines Caucasians. Our results suggested that rs10823108 AA genotype within SIRT1 has significant associations with about a 0.60-fold decreased risk of DN (OR = 0.60,95%CI:0.38–0.97) than GG genotype carriers, even after adjustments for age, gender, duration of diabetes, BMI, HbA1C,total cholesterol, triglycerides, HDL-C, LDL-C, history of Hypertension, DM family history, smoking and drinking status (OR = 0.57, 95%CI: 0.34–0.94), which is similar with the result in Japanese population^17^. We can’t see any significant difference between rs4746720 and rs3818292 SNPs and diabetic nephropathy in univariate analysis and multivariate analysis, which is different with the results in Japanese, which may be due to ethnic differences and genetic backgrounds. In the next stratified analysis, rs3818292 AG + GG genotypes had a significant increased risk of T2DN in subjects with TC level > 5 (OR: 2.11; 95%CI: 1.06–4.21).

A previous study has shown that rs17446614 and rs2721068 displayed significant associations with impaired glucose tolerance and beta-cell dysfunction^22^. In our study, we investigated the role of FOXO1 variants (rs17446614 and rs2721068) in T2DN for the first time. It is clear that FOXO1 rs17446614 GA genotype (OR = 1.77, 95%CI: 1.33–2.35) and AA genotype (OR = 2.32, 95%CI: 1.25–4.34) could increase the risk of suffering from T2DN when compared to GG genotype carriers. No significant association was found for rs2721068 in both univariate analysis and multivariate analysis. However, in the stratified analysis, TC + CC genotypes of rs2721068 had a significantly decreased risk of T2DN among male subjects (OR: 0.47, 95%CI: 0.25–0.87), younger subjects (OR: 0.35; 95%CI: 0.17–0.71), subjects with BMI ≥ 24 (OR: 0.46; 95%CI: 0.26–0.79), LDL-C ≤ 3.5 (OR: 0.61; 95%CI: 0.38–0.98) and smoking (OR: 0.14; 95%CI: 0.03–0.64). In order to find more credible evidence on the association between FOXO1polymorphisms and T2DN risk, further functional studies on different populations are required.

Haplotype was accepted as a group of correlated SNPs that are located in the same homologous chromosome, and passed on to descendants together as a whole^23^. Haplotype contained multiple SNPs information, and the statistical power of haplotype analysis was stronger than analysis of single SNP^24^. Furthermore, we detected that G_rs17446614_T_rs2721068_ (OR:0.77, 95%CI:0.63–0.93) and G_rs17446614_C_rs2721068_ (OR:0.78, 95%CI:0.65–0.93) within FOXO1 could reduce the risk of DN in T2DM patients. Therefore, our results suggest that the SNPs within SIRT1 and FOXO1 might be involved in the process of diabetic nephropathy in Henan Han Chinese population. In this study, the gene–reproductive factors interaction on T2DN susceptibility was examined by using a MDR method. Anemically significant interaction was found for rs1746614, BMI and duration of diabetes (OR:4.01, 95%CI:3.02–5.31). To our knowledge, this is the first study to examine the effect of FOXO1 polymorphisms on T2DN risk with an emphasis on the gene-environment factors interaction.

Although, the mechanism by which the SIRT1 polymorphism contributes to conferring susceptibility to diabetic nephropathy remains to be elucidated, combining the present finding with a previous report, SIRT1 and FOXO1 may be considered a good new candidate gene for diabetic nephropathy. The effects of SIRT1 polymorphisms on susceptibility to DN might be mediated by differences in the metabolic state among individuals, including glycemic control, obesity, blood pressure, respectively^25–27^. The evidence in Wild-type (C57BL/6 J) and SIRT1 transgenic mice (C57BL/6-Actb^tm3.1(Sirt1)Npa^/J) showed SIRT1 mediated protection against renal and retinal injury in DN^28^. FOXO1, as an important target for insulin and growth factor signaling, has been reported to play key roles in regulating metabolism, cell proliferation, cell cycle, apoptosis and oxidative stress response by regulating the expression of functionally important target genes^29, 30^. It is shown that GWAS in Pima Indians identified variation within FOXO1A that modestly associated with early-onset T2DN. Common variation in FOXO1A may modestly affect risk for T2D and obesity in American Indians^31^. Interestingly, our previous studies have confirmed that the transcriptional activity of FOXO1 was decreased in both renal cortex of DN rats and MCs cultured under high-glucose (HG) conditions, which indicated that FOXO1 may be considered a good new candidate gene for diabetic nephropathy^14, 15^.

Our study has several notable strengths. First, although the role of SIRT1 and FOXO1 in multiple diseases has been reported, this is the first study investigating genetic variation of FOXO1 and SIRT1 in relation to T2DN susceptibility. Second, in order to draw a convincing conclusion, only patients who had suffered T2DM for more than 8 years and no diabetic retinopathy were eligible to participate in the control group, because the duration of DM plays a very important role in the development of DN, and diabetic retinopathy has a complicated relationship with DN^32^. To increase comparability between DN cases and controls, the DN cases and the controls were much more homogeneous in terms of gender, age, and durations of diabetes, and family history, after excluding subjects with extreme characteristics. Third, our genotyping results were accurate and reliable, since more than 30% of the samples were genotyped in duplicate. 10% of the samples were randomly selected from both cases and controls to confirm the genotyping results, and the result of confirmation were found to be 100% concordant.

Some limitations of this study should also be noted. One limitation was the limited size sample in this study, which might be the reason why we didn’t see any statistical significance in gene-gene interaction analysis. Secondly, although we identified two novel SNPs that are susceptibility variants of DN in a Han Chinese population in Henan, screening more loci within SIRT1 gene and FOXO1 gene to further clarify the association between them and DN is clearly warranted. In order to validate our findings, further research needs to be conducted, including further functional evaluation with other ethnic populations and animal experiments.

BMI might be a factor in affecting SIRT-1 activation and reversing epigenetic metabolic imprinting or silencing. In the recent report, the clinical evidence also support the relationship of BMI (Epicardial fat thickness) and circulating SIRT1^33^. In our study, the data of BMI was 26.19 ± 2.55 kg/m^2^ in case group while 25.25 ± 2.74 kg/m^2^ in control group. Interestingly, their findings are consistent with our results. Of couse, more evidence will be confrimed in animal experiments. Additionally, the activation of SIRT1 might be associated with Zinc levels. In the study of Sheline C.T., he/she found that SIRT1-mediated NAD^+^ loss in Zn^2+^, STZ, or cytokine toxicities of MIN6, and in NOD or DM animal models^34^. These findings indicated that β-cell Zn^2+^ levels play a regulator in DM and its vascular complications, including DN. The interesting evidence need to be provided in our following experiments.

Despite the several limitations mentioned above, to the best of our knowledge, our data first investigated the association between SIRT1 and FOXO1 gene and T2DN in Chinese Han population. This study has made a valuable contribution to draw a plausible link between SIRT1 and FOXO1 gene polymorphisms and DN. However, replication in additional DM patient cohorts, case control studies, and further functional work to dissect out the role of SIRT1 and FOXO1 polymorphisms *in vitro* conditions, are clearly needed. Furthermore, on the basis of the current genotyping and clinical staging, prospective studies in the disease outcome in different stages, especially in patients with early diabetic nephropathy group, further verify the role of each gene locus in diabetic nephropathy, which will be more interesting and meaningful.

We identified two novel SNPs (rs10823108 in SIRT1, rs17446614 in FOXO1) and two haplotypes within FOXO1 gene (defined by GT and GC), that are susceptibility variants of DN in a Han Chinese population in Henan. The findings reveal that SIRT1/FOXO1 may involve in the initiation and development of DN in Han Chinese patients with type 2 diabetes. Our results can provide new insights into the etiology of DN.

## Materials and Methods

### Study subjects

A case-control study was performed in Han Chinese population located in Henan province. Individuals with type 2 diabetes and aged over 20 years were recruited from June 2012 to Sep 2013 using the American Diabetes Association (ICD-9-CM, Diagnosis code 250) criteria for diagnosis of type 2 diabetes. A total of 1066 type 2 diabetes subjects, recruited from the first affiliated hospital of Zhengzhou University, located in the middle part of China, were involved in this study. All patients with type 2 diabetes were of Han Chinese origin. Subjects who tested positive for anti-glutamic acid decarboxylase (GAD) antibodies and those diagnosed with mitochondrial disease (mitochondrial myopathy, encephalopathy, lactic acidosis, and stroke-like episodes [MELAS]) or maturity-onset diabetes of the young (MODY), gestational diabetes and secondary diabetes were not included.

Diabetic nephropathy was determined by estimated 24 h albumin excretion rates (AERs) or urinary albumin-to-creatinine ratios (ACR)^35, 36^. AERs or ACR were measured twice, and the mean value was recorded for each patient. Patients having microalbuminuria, indicated 30 mg/24 h ≤ AERs < 300 mg/24 h or 30 mg/g ≤ ACR < 300 mg/g, and with background or greater diabetic retinopathy, or proteinuria indicated AERs ≥ 300 mg/24 h or ACR ≥ 300 mg/g, or ESRD diagnosed at least 5 years before initiating renal replacement therapy, were diagnosed with diabetic nephropathy (n = 653). Patients with no diabetic retinopathy, and normal albuminuria, indicated AERs < 30 mg/24 h or ACR < 30 mg/g, and a long T2DM duration over 8 years were selected as a control group (n = 413). All the patients participating in this study provided written informed consent, and the study protocol was approved by the ethics committees of the first affiliated hospital of Zhengzhou University. We confirmed that all experiments were performed in accordance with relevant guidelines and regulations.

### Measurements

Family history was defined as the presence of T2DM in either a parent or a sibling; smoking was defined as having smoked more than 1 cigarette every day and at least one year in one’s lifetime; similarly, drinking was defined as alcohol intake more than once per week. Anthropometric measurements included weight (kg); height (cm); BMI was calculated as weight divided by squared height. The fasting serum biochemical profile includes total cholesterol (mmol/L); LDL (mmol/L); HDL (mmol/L), and triglycerides (mmol/L), were determined by using cobas c8000 clinical Chemistry System (Roche, German). After an early morning spot urine sample collection, urinary albumin and creatinine concentrations were measured by Afinion AS100 Analyzer and the urine albumin-to-creatinine ratio (UACR) was defined as the urinary albumin value divided by the creatinine concentration (mg/g).

### SNP selection and genotyping

Reference sequences of SIRT1 and FOXO1 were identified with National Center for Biotechnology Information (NCBI) database (http://www.ncbi.nlm.nih.gov/). Haplotype-tagging SNPs (htSNPs) were used to analyze the SIRT1 and FOXO1 polymorphisms globally. htSNPs selection was conducted with the pairwise option of the Haploview software 4.2, minor allele frequency (MAF) > 0.1 and the pairwise correlation was set as r^2^ > 0.8, based on the Chinese Han population data of HapMap (HapMap Data Rel 28 Phase II + III, August 10, on NCBI B36 assembly, dbSNP b126). In summary, five htSNPs (rs3818292 A > G, rs4746720 T > C, rs10823108 G > A in SIRT1 and rs2721068 T > C, rs17446614 G > A in FOXO1) that captured most of the known common SNPs located in the chromosome locus transcribed into SIRT1 and FOXO1 and its flanking region (2000 bp upstream and 2000 bp downstream, respectively) were selected.

DNA was extracted from a peripheral blood sample using a QIAamp (Qiagen, Germany) kit, and analyzed by electrophoresis in 0.8% agarose gels stained with ethidium bromide and visualized in a Gel Doc 2000 (BIORAD CA, USA). DNA concentration was determined using a VICTOR3 1420 spectrophotometer (Perkin–Elmer, Germany). The five htSNPs were genotyped with restriction fragment length polymorphism method (PCR-RFLP). Primers and restriction endonucleases were shown in Table 7. The volume of PCR was 15 μl, which contained 7.5 μl 2 × Tap PCR Master Mix, 0.8 μM each primer, 50 ng DNA, and 5.7 μl deionized water. The PCR profile included an initial melting step of 95 °C for 5 min, followed by 35 cycles of denaturation at 95 °C for 30 s, annealing at 59 °C, 59 °C, 59 °C, 57 °C, and 57 °C for rs3818292, rs4746720, rs10823108, rs2721068, and rs17446614, respectively for 45 s and extension at 72 °C for 1 min and a final extension step of 72 °C for 10 min. PCR products were digested for 3–16 h at 37 °C by VspI, Hpy1881, Hpy1881, TspRI, and CviJI (Fermentas) for rs3818292, rs4746720, rs10823108, rs2721068, and rs17446614, respectively. The digested PCR products were separated on 3% agarose gel electrophoresis and stained with ethidium bromide. For each SNP in the groups of study, a concordance of 100% was observed when 30–50% of the samples were genotyped in duplicate. 10% samples were randomly selected from both cases and controls to confirm the genotyping results. The direct sequencing (BGI Sequencing, Beijing) was also utilized for the genotype confirmation, and the result of confirmation were found to be 100% concordant.

**Table 7:** Detail of PCR Primers and restriction endonucleases primers.

SNP	Location	Chr:position	MAF^a^	Endonuclease	Primers
rs3818292 A > G	SIRT1,Intron	Chr10:67907144	G = 0.1272	VspI	F: GACAATTCCAGCCATCTCT
					R: CTCACGCCTGTAATCCTAG
rs4746720 T > C	SIRT1,Intron	Chr10:67917073	C = 0.1156	Hpy1881	F: AGGGAACAGCTAATCTAGACCA
					R: AAAGTAAAGACAACCGAGTGCT
rs10823108 G > A	SIRT1,Intron	Chr10:67900736	A = 0.1272	Hpy1881	F: TCTCAGCCTCCCAAGTAG
					R: AGTAGTAACCAGCAGTCATC
rs17446614 G > A	FOXO1,Intron	Chr13:40565740	A = 0.1767	CviJI	F: CACAGCACCAACACCATAG
					R: GACAGCCTCACCTTAGTATT
rs2721068 T > C	FOXO1,Intron	Chr13:40565575	T = 0.4856	TspRI	F: AGAGCCACTTAATAGGATGAC
					R: ACCTTTGTCTGTCTTCTTGTACAC

^a^based on the Chinese Han population data of HapMap.

### Statistical analysis

Difference in the distribution of demographic characteristics and genotype frequencies between cases and controls were evaluated using the χ^2^-test (Chi-square test). The associations between rs3818292, rs4746720, rs10823108, rs2721068, rs17446614 genotypes and risk of T2DN were estimated by computing the odds ratio (ORs) and their 95% confidence interval(CI). Hardy–Weinberg equilibrium (HWE) for the genotype distribution of each SNP was tested by a goodness-of-fit Chi squared test to compare the observed genotype frequencies with the expected ones among the control subjects. The data were further stratified by demographic characteristics and biochemical factors to evaluate the stratum variable-related ORs among the five SNPs. MDR methods were used to detect the gene-environment factors interaction. The online SHEsis (http://analysis.bio-x.cn/myAnalysis.php) was taken for Haplotype Analysis. Statistical analysis was performed by using SPSS 17.0 and SAS 9.1 software package. All tests were two sided and a P value < 0.05 was considered to be statistically significant.

## References

[1] Wanner C (2016). Empagliflozin and Progression of Kidney Disease in Type 2 Diabetes. N Engl J Med..

[2] Al-Rubeaan K (2017). Assessment of the diagnostic value of different biomarkers in relation to various stages of diabetic nephropathy in type 2 diabetic patients. Sci Rep..

[3] Collins AJ (2012). US Renal Data System 2011 Annual Data Report. Am J Kidney Dis..

[4] Yang YZ (2017). Incidence, Development, and Prognosis of Diabetic Kidney Disease in China: Design and Methods. Chin Med J (Engl)..

[5] Zhang L (2016). Trends in Chronic Kidney Disease in China. N Engl J Med..

[6] Jiao F (2016). Effectiveness of the multidisciplinary Risk Assessment and Management Program for Patients with Diabetes Mellitus (RAMP-DM) for diabetic microvascular complications: A population-based cohort study. Diabetes Metab..

[7] Pezzolesi MG, Krolewski AS (2013). The Genetic Risk of Kidney Disease in Type 2 Diabetes. Med Clin North Am..

[8] Wu L (2015). Activation of FoxO1/ PGC-1αprevents mitochondrial dysfunction and ameliorates mesangial cell injury in diabetic rats. Mol Cell Endocrinol..

[9] Ponugoti B, Dong GY, Graves DT (2012). Role of Forkhead Transcription Factors in Diabetes-Induced Oxidative Stress. Exp Diabetes Res..

[10] Wu L, Zhang Y, Ma X, Zhang N, Qin G (2012). The effect of resveratrol on FoxO1 expression in kidneys of diabetic nephropathy rats. Mol Biol Rep..

[11] Dong P (2014). MiR-15a/b promote adipogenesis in porcine pre-adipocyte via repressing FoxO1. Acta Biochim Biophys Sin (Shanghai)..

[12] Jing E, Gesta S, Kahn CR (2017). SIRT2 regulates adipocyte differentiation through FoxO1 acetylation/deacetylation. Cell Metab..

[13] Chang HC, Guarente L (2014). SIRT1 and other sirtuins in metabolism. Trends Endocrin Met..

[14] Kume S, Kitada M, Kanasaki K, Maegawa H, Koya D (2013). Anti-aging molecule, Sirt1: a novel therapeutic target for diabetic nephropathy. Arch Pharm Res..

[15] Kitada M, Kume S, Takeda-Watanabe A, Kanasaki K, Koya D (2013). Sirtuins and renal diseases: relationship with aging and diabetic nephropathy. Clin Sci..

[16] Li, W. *et al*. FoxO1 Promotes Mitophagy in the Podocytes of Diabetic Male Mice via the PINK1/Parkin Pathway. *Endocrinology*. doi:10.1210/en.2016-1970 (2017). 10.1210/en.2016-1970 28505239

[17] Maeda S (2015). Association between single nucleotide polymorphisms within genes encoding sirtuin families and diabetic nephropathy in Japanese subjects with type 2 diabetes. Clin Exp Nephrol..

[18] Böttcher Y (2007). A SNP haplotype of the forkhead transcription factor FOXO1A gene may have a protective effect against type 2 diabetes in German Caucasians. Diabetes Metab..

[19] Sasso FC (2016). Moderate-intensity statin therapy seems ineffective in primary cardiovascular prevention in patients with type 2 diabetes complicated by nephropathy. A multicenter prospective 8 years follow up study. Cardiovasc Diabetol..

[20] Seaquist ER, Goetz FC, Rich S, Barbosa J (1989). Familial clustering of diabetic kidney disease. Evidence for genetic susceptibility to diabetic nephropathy. N Engl J Med..

[21] Todd JN (2015). Genetic Evidence for a Causal Role of Obesity in Diabetic Kidney Disease. Diabetes..

[22] Mussig K (2009). Association of common genetic variation in the FOXO1 gene with beta-cell dysfunction, impaired glucose tolerance, and type 2 diabetes. J Clin Endocrinol Metab..

[23] Gabriel SB (2002). The structure of haplotype blocks in the human genome. Science..

[24] Akey J, Jin L, Xiong MM (2001). Haplotypes vs single marker linkage disequilibrium tests: what do we gain?. Eur J Hum Genet..

[25] Navarro-Gonzalez JF, Mora-Fernandez C, Muros de Fuentes M, Garcia-Perez J (2011). Inflammatory molecules and pathways in the pathogenesis of diabetic nephropathy. Nat Rev Nephrol..

[26] Huang K (2015). Polydatin promotes Nrf2-ARE anti-oxidative pathway through activating Sirt1 to resist AGEs-induced upregulation of fibronetin and transforming growth factor-β1 in rat glomerular messangial cells. Mol Cell Endocrinol..

[27] Kume S (2007). SIRT1 inhibits transforming growth factor beta-induced apoptosis in glomerular mesangial cells via Smad7 deacetylation. J Biol Chem..

[28] Mortuza R, Feng B, Chakrabarti S (2015). SIRT1 reduction causes renal and retinal injury in diabetes through endothelin 1 and transforming growth factor β1. J Cell Mol Med..

[29] Du M (2016). Overexpression of FOXO1 ameliorates the podocyte epithelial-mesenchymal transition induced by high glucose *in vitro* and *in vivo*. Biochem Biophys Res Commun..

[30] Wang B (2011). Bufalin Inhibits Platelet-Derived Growth Factor-BB-Induced Mesangial Cell Proliferation through Mediating Cell Cycle Progression. Biol Pharm Bull..

[31] Muller YL (2015). Assessing FOXO1Aas a Potential Susceptibility Locus for Type 2 Diabetes and Obesity in American Indians. Obesity..

[32] He F, Xia X, Wu XF, Yu XQ, Huang FX (2013). Diabetic retinopathy in predicting diabetic nephropathy in patients with type 2 diabetes and renal disease: a meta-analysis. Diabetologia..

[33] Mariani S (2016). Circulating SIRT1 inversely correlates with epicardial fat thickness in patients with obesity. Nutr Metab Cardiovasc Dis..

[34] Sheline CT (2012). Involvement of SIRT1 in Zn2 + , Streptozotocin, Non-Obese Diabetic, and Cytokine-Mediated Toxicities of β-cells. J Diabetes Metab..

[35] Everett CJ, Thompson OM, Dismuke CE (2017). Exposure to DDT and diabetic nephropathy among Mexican Americans in the 1999-2004 National Health and Nutrition Examination Survey. Environ Pollut..

[36] Kitaoka K, Takenouchi A, Tsuboi A, Fukuo K, Kazumi T (2016). Association of Postbreakfast Triglyceride and Visit-to-Visit Annual Variation of Fasting Plasma Glucose with Progression of Diabetic Nephropathy in Patients with Type 2 Diabetes. J Diabetes Res..

